# 2-[(2-Bromo­phen­yl)imino­meth­yl]-6-methyl­phenol

**DOI:** 10.1107/S1600536810043072

**Published:** 2010-10-30

**Authors:** Aslı Tosyalı Karadağ, Şehriman Atalay, Hasan Genç

**Affiliations:** a Department of Physics, Faculty of Arts and Sciences, Ondokuz Mayıs University, Kurupelit, TR-55139 Samsun, Turkey; b Department of Chemistry, Faculty of Arts and Sciences, Yüzüncü Yıl Univercity, 65250 Van, Turkey

## Abstract

In the title compound, C_14_H_12_BrNO, is a Schiff base which adopts the phenol–imine tautomeric form in the solid state. The dihedral angle between the two aromatic rings is 34.26 (9)° and an intra­molecular O—H⋯N hydrogen bond generates an *S*(6) ring.

## Related literature

For Schiff bases and their applications, see: Calligaris *et al.* (1972 ▶); Singh *et al.* (1975 ▶). For a related structure, see: Temel *et al.* (2007 ▶).
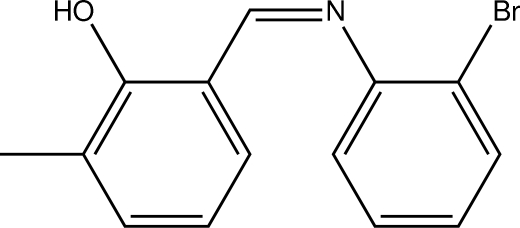


## Experimental

### 

#### Crystal data

C_14_H_12_BrNO
*M* *_r_* = 290.16
Orthorhombic, 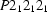

*a* = 7.9407 (4) Å
*b* = 11.6754 (8) Å
*c* = 13.1960 (6) Å
*V* = 1223.41 (12) Å^3^
*Z* = 4
Mo *K*α radiation
μ = 3.34 mm^−1^
*T* = 296 K
0.47 × 0.39 × 0.24 mm

#### Data collection

Stoe IPDS 2 diffractometer
Absorption correction: integration (*X-RED32*; Stoe & Cie, 2002 ▶) *T*_min_ = 0.358, *T*_max_ = 0.525
21115 measured reflections
2929 independent reflections
2427 reflections with *I* > 2σ(*I*)
*R*_int_ = 0.067

#### Refinement

*R*[*F* ^2^ > 2σ(*F* ^2^)] = 0.035
*wR*(*F* ^2^) = 0.080
*S* = 1.07
2929 reflections
160 parameters
H atoms treated by a mixture of independent and constrained refinement
Δρ_max_ = 0.64 e Å^−3^
Δρ_min_ = −0.27 e Å^−3^
Absolute structure: Flack (1983 ▶), 1229 Friedel pairs
Flack parameter: −0.003 (10)

### 

Data collection: *X-AREA* (Stoe & Cie, 2002 ▶); cell refinement: *X-AREA*; data reduction: *X-RED32* (Stoe & Cie, 2002 ▶); program(s) used to solve structure: *SHELXS97* (Sheldrick, 2008 ▶); program(s) used to refine structure: *SHELXL97* (Sheldrick, 2008 ▶); molecular graphics: *ORTEP-3 for Windows* (Farrugia, 1997 ▶); software used to prepare material for publication: *WinGX* (Farrugia, 1999 ▶).

## Supplementary Material

Crystal structure: contains datablocks I, global. DOI: 10.1107/S1600536810043072/bt5384sup1.cif

Structure factors: contains datablocks I. DOI: 10.1107/S1600536810043072/bt5384Isup2.hkl

Additional supplementary materials:  crystallographic information; 3D view; checkCIF report

## Figures and Tables

**Table 1:** Hydrogen-bond geometry (Å, °)

*D*—H⋯*A*	*D*—H	H⋯*A*	*D*⋯*A*	*D*—H⋯*A*
O1—H1⋯N1	0.89 (4)	1.81 (3)	2.611 (3)	149 (3)

**Fig. 1.**
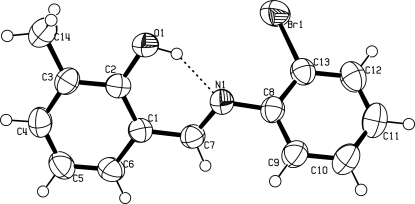
The molecular structure of the title compound, showing the atom-numbering scheme. Displacement ellipsoids are drawn at the 50% probability.

## supplementary crystallographic information

### Comment

Schiff bases have been used extensively as ligands in the field of coordination chemistry (Calligaris *et al.*, 1972). Schiff bases derived from aromatic amines and aromatic aldehydes have a wide variety of applications in many fields, *e.g.*, biological, inorganic and analytical chemistry (Singh *et al.*, 1975).

Schiff base compounds show photochromism and thermochromism in the solid state by proton transfer from the hydroxyl O atom to the imine N atom.

The overall behaviour of these compounds has been ascribed to a proton-transfer reaction between a phenol-imine and a keto-amine tautomer. In solution, the existence of this tautomerism, which depends on the formation of intramolecular hydrogen bonds, is possible.

X-ray investigation of the title compound, (I), has indicated that the phenol-imine tautomer is favoured over the keto-amine tautomer. Bond lengths (Fig. 1) C2—O1 [1.364 (3) Å], C7—N1 [1.285 (3) Å], C1—C7 [1.451 (4) Å] and C1—C2 [1.403 (4) Å]. The C2—O1 bond length of 1.364 (3) Å indicates a single-bond character, whereas the C7—N1 bond length of 1.285 (3) Å indicates a high degree of double-bond character. Similar results were observed for (E)-3-[(2- fluorophenylimino)methyl]benzene-1,2-diol [C—O = 1.354 (19) Å, C—N = 1.285 (2) Å; Temel *et al.*, 2007].

N···H—O hydrogen bond generate an S(6) ring motif  (Fig. 1).

### Experimental

The compound 2-[(2-Bromophenylimino) methyl]-6- methylphenol was prepared by reflux a mixture of a solution containing 3-Methylsalicylaldehyde (0.05 g 0.36 mmol) in 20 ml e thanol and a solution containing 2-Bromoaniline(0.062 g 0.36 mmol) in 20 ml e thanol. The reaction mixture was stirred for 1 hunder reflux. The crystals of 2-[(2-Bromophenylimino) methyl]-6- methylphenol suitable for X-ray analysis were obtained from ethylalcohol by slow evaporation (yield % 63; m.p.418–420 K).

### Refinement

H atoms were positioned geometrically with distances 0.93 Å for aromatic C—H, 0.97 Å for methylene C—H, 0.86 Å for O—H hydroxyl group and refined a riding model with *U*_iso_(H) = 1.2*U*_eq_(C,O).

### Crystal data

**Table:**

C_14_H_12_BrNO	*F*(000) = 584
*M**_r_* = 290.16	*D*_x_ = 1.575 Mg m^−^^3^
Orthorhombic, *P*2_1_2_1_2_1_	Mo *K*α radiation, λ = 0.71073 Å
Hall symbol: P 2ac 2ab	Cell parameters from 23647 reflections
*a* = 7.9407 (4) Å	θ = 2.3–28.0°
*b* = 11.6754 (8) Å	µ = 3.34 mm^−^^1^
*c* = 13.1960 (6) Å	*T* = 296 K
*V* = 1223.41 (12) Å^3^	Prism, yellow
*Z* = 4	0.47 × 0.39 × 0.24 mm

### Data collection

**Table:**

Stoe IPDS 2 diffractometer	2929 independent reflections
Radiation source: fine-focus sealed tube	2427 reflections with *I* > 2σ(*I*)
graphite	*R*_int_ = 0.067
Detector resolution: 6.67 pixels mm^-1^	θ_max_ = 28.0°, θ_min_ = 2.3°
rotation method scans	*h* = −10→10
Absorption correction: integration (*X-RED32*; Stoe & Cie, 2002)	*k* = −15→15
*T*_min_ = 0.358, *T*_max_ = 0.525	*l* = −17→17
21115 measured reflections	

### Refinement

**Table:**

Refinement on *F*^2^	Hydrogen site location: inferred from neighbouring sites
Least-squares matrix: full	H atoms treated by a mixture of independent and constrained refinement
*R*[*F*^2^ > 2σ(*F*^2^)] = 0.035	*w* = 1/[σ^2^(*F*_o_^2^) + (0.0416*P*)^2^ + 0.0403*P*] where *P* = (*F*_o_^2^ + 2*F*_c_^2^)/3
*wR*(*F*^2^) = 0.080	(Δ/σ)_max_ = 0.001
*S* = 1.07	Δρ_max_ = 0.64 e Å^−^^3^
2929 reflections	Δρ_min_ = −0.27 e Å^−^^3^
160 parameters	Extinction correction: *SHELXL97* (Sheldrick, 2008), Fc^*^=kFc[1+0.001xFc^2^λ^3^/sin(2θ)]^-1/4^
0 restraints	Extinction coefficient: 0.0200 (16)
Primary atom site location: structure-invariant direct methods	Absolute structure: Flack (1983), 1229 Friedel pairs
Secondary atom site location: difference Fourier map	Flack parameter: −0.003 (10)

### Special details

**Table:**

Geometry. All e.s.d.'s (except the e.s.d. in the dihedral angle between two l.s. planes) are estimated using the full covariance matrix. The cell e.s.d.'s are taken into account individually in the estimation of e.s.d.'s in distances, angles and torsion angles; correlations between e.s.d.'s in cell parameters are only used when they are defined by crystal symmetry. An approximate (isotropic) treatment of cell e.s.d.'s is used for estimating e.s.d.'s involving l.s. planes.
Refinement. Refinement of *F*^2^ against ALL reflections. The weighted *R*-factor *wR* and goodness of fit *S* are based on *F*^2^, conventional *R*-factors *R* are based on *F*, with *F* set to zero for negative *F*^2^. The threshold expression of *F*^2^ > σ(*F*^2^) is used only for calculating *R*-factors(gt) *etc*. and is not relevant to the choice of reflections for refinement. *R*-factors based on *F*^2^ are statistically about twice as large as those based on *F*, and *R*- factors based on ALL data will be even larger.

### Fractional atomic coordinates and isotropic or equivalent isotropic displacement parameters (Å^2^)

**Table:**

	*x*	*y*	*z*	*U*_iso_*/*U*_eq_	
H1	0.744 (4)	0.652 (3)	0.652 (3)	0.062 (9)*	
Br1	0.79638 (5)	0.84452 (3)	0.81971 (3)	0.07572 (15)	
C4	0.7065 (4)	0.5259 (2)	0.3545 (2)	0.0582 (7)	
H4	0.6400	0.4945	0.3036	0.070*	
N1	0.9650 (3)	0.68078 (17)	0.67303 (16)	0.0487 (5)	
C7	1.0181 (3)	0.6620 (2)	0.58252 (19)	0.0477 (5)	
H7	1.1302	0.6768	0.5669	0.057*	
C5	0.8731 (4)	0.5467 (3)	0.3351 (2)	0.0609 (7)	
H5	0.9181	0.5292	0.2719	0.073*	
C1	0.9072 (3)	0.6182 (2)	0.50413 (18)	0.0452 (5)	
O1	0.6641 (2)	0.62056 (19)	0.61369 (15)	0.0596 (5)	
C2	0.7361 (3)	0.5966 (2)	0.52228 (18)	0.0457 (6)	
C10	1.3450 (4)	0.7113 (3)	0.8347 (3)	0.0666 (8)	
H10	1.4553	0.6848	0.8380	0.080*	
C13	1.0198 (4)	0.7883 (2)	0.8254 (2)	0.0527 (6)	
C9	1.2424 (4)	0.6774 (2)	0.7555 (2)	0.0569 (7)	
H9	1.2843	0.6284	0.7060	0.068*	
C6	0.9732 (4)	0.5931 (3)	0.40857 (19)	0.0568 (7)	
H6	1.0860	0.6080	0.3949	0.068*	
C3	0.6334 (4)	0.5500 (2)	0.4475 (2)	0.0503 (6)	
C8	1.0769 (3)	0.7162 (2)	0.7491 (2)	0.0482 (6)	
C11	1.2857 (5)	0.7834 (3)	0.9084 (3)	0.0683 (8)	
H11	1.3557	0.8059	0.9613	0.082*	
C14	0.4513 (4)	0.5289 (3)	0.4673 (3)	0.0710 (8)	
H14A	0.4396	0.4770	0.5233	0.106*	
H14B	0.4003	0.4960	0.4081	0.106*	
H14C	0.3967	0.6000	0.4832	0.106*	
C12	1.1230 (5)	0.8226 (3)	0.9041 (2)	0.0626 (8)	
H12	1.0823	0.8718	0.9538	0.075*	

### Atomic displacement parameters (Å^2^)

**Table:**

	*U*^11^	*U*^22^	*U*^33^	*U*^12^	*U*^13^	*U*^23^
Br1	0.0655 (2)	0.0941 (2)	0.06760 (19)	0.01958 (17)	0.00516 (17)	−0.00921 (17)
C4	0.0647 (17)	0.0583 (15)	0.0515 (13)	0.0010 (15)	−0.0149 (14)	−0.0035 (11)
N1	0.0488 (11)	0.0516 (11)	0.0456 (10)	−0.0009 (8)	−0.0020 (10)	−0.0013 (10)
C7	0.0435 (12)	0.0506 (12)	0.0490 (13)	−0.0016 (12)	0.0018 (11)	0.0009 (12)
C5	0.0707 (18)	0.0695 (17)	0.0425 (14)	0.0056 (15)	0.0005 (13)	−0.0047 (13)
C1	0.0471 (13)	0.0462 (13)	0.0422 (11)	0.0007 (10)	0.0007 (11)	0.0048 (9)
O1	0.0456 (10)	0.0832 (14)	0.0500 (10)	−0.0008 (9)	0.0042 (9)	−0.0049 (9)
C2	0.0469 (14)	0.0450 (12)	0.0450 (12)	0.0035 (10)	0.0006 (10)	0.0046 (10)
C10	0.0527 (16)	0.0665 (17)	0.081 (2)	−0.0055 (13)	−0.0152 (15)	−0.0016 (16)
C13	0.0576 (15)	0.0524 (13)	0.0482 (13)	−0.0044 (12)	0.0004 (14)	0.0009 (13)
C9	0.0486 (15)	0.0564 (16)	0.0657 (16)	−0.0019 (11)	−0.0052 (12)	−0.0064 (12)
C6	0.0542 (15)	0.0695 (17)	0.0467 (14)	0.0024 (13)	0.0078 (13)	0.0004 (13)
C3	0.0466 (13)	0.0507 (13)	0.0536 (14)	0.0009 (11)	−0.0083 (12)	0.0049 (11)
C8	0.0492 (14)	0.0464 (13)	0.0489 (13)	−0.0062 (11)	−0.0030 (12)	0.0016 (11)
C11	0.073 (2)	0.0618 (17)	0.0697 (18)	−0.0122 (16)	−0.0224 (18)	0.0005 (14)
C14	0.0507 (17)	0.083 (2)	0.079 (2)	−0.0072 (15)	−0.0082 (16)	−0.0021 (18)
C12	0.077 (2)	0.0556 (17)	0.0554 (15)	−0.0096 (15)	−0.0013 (15)	−0.0058 (13)

### Geometric parameters (Å, °)

**Table:**

Br1—C13	1.893 (3)	C10—C11	1.370 (5)
C4—C5	1.370 (5)	C10—C9	1.383 (4)
C4—C3	1.386 (4)	C10—H10	0.9300
C4—H4	0.9300	C13—C12	1.382 (4)
N1—C7	1.285 (3)	C13—C8	1.388 (4)
N1—C8	1.402 (3)	C9—C8	1.392 (4)
C7—C1	1.451 (4)	C9—H9	0.9300
C7—H7	0.9300	C6—H6	0.9300
C5—C6	1.366 (4)	C3—C14	1.490 (4)
C5—H5	0.9300	C11—C12	1.372 (5)
C1—C6	1.397 (4)	C11—H11	0.9300
C1—C2	1.403 (4)	C14—H14A	0.9600
O1—C2	1.364 (3)	C14—H14B	0.9600
O1—H1	0.89 (4)	C14—H14C	0.9600
C2—C3	1.390 (4)	C12—H12	0.9300
			
C5—C4—C3	122.3 (3)	C10—C9—H9	119.7
C5—C4—H4	118.9	C8—C9—H9	119.7
C3—C4—H4	118.9	C5—C6—C1	120.4 (3)
C7—N1—C8	120.5 (2)	C5—C6—H6	119.8
N1—C7—C1	121.6 (2)	C1—C6—H6	119.8
N1—C7—H7	119.2	C4—C3—C2	117.5 (3)
C1—C7—H7	119.2	C4—C3—C14	121.8 (3)
C6—C5—C4	120.0 (3)	C2—C3—C14	120.7 (3)
C6—C5—H5	120.0	C13—C8—C9	117.4 (3)
C4—C5—H5	120.0	C13—C8—N1	119.4 (3)
C6—C1—C2	118.7 (2)	C9—C8—N1	123.1 (2)
C6—C1—C7	119.3 (3)	C10—C11—C12	119.9 (3)
C2—C1—C7	122.0 (2)	C10—C11—H11	120.0
C2—O1—H1	107 (2)	C12—C11—H11	120.0
O1—C2—C3	117.5 (2)	C3—C14—H14A	109.5
O1—C2—C1	121.3 (2)	C3—C14—H14B	109.5
C3—C2—C1	121.2 (2)	H14A—C14—H14B	109.5
C11—C10—C9	120.7 (3)	C3—C14—H14C	109.5
C11—C10—H10	119.7	H14A—C14—H14C	109.5
C9—C10—H10	119.7	H14B—C14—H14C	109.5
C12—C13—C8	121.8 (3)	C11—C12—C13	119.5 (3)
C12—C13—Br1	119.0 (2)	C11—C12—H12	120.2
C8—C13—Br1	119.2 (2)	C13—C12—H12	120.2
C10—C9—C8	120.6 (3)		
			
C8—N1—C7—C1	176.0 (2)	C1—C2—C3—C4	0.0 (4)
C3—C4—C5—C6	−0.2 (5)	O1—C2—C3—C14	0.7 (4)
N1—C7—C1—C6	−176.4 (3)	C1—C2—C3—C14	−179.2 (3)
N1—C7—C1—C2	1.4 (4)	C12—C13—C8—C9	1.1 (4)
C6—C1—C2—O1	−179.3 (2)	Br1—C13—C8—C9	179.5 (2)
C7—C1—C2—O1	2.9 (4)	C12—C13—C8—N1	177.7 (2)
C6—C1—C2—C3	0.6 (4)	Br1—C13—C8—N1	−4.0 (3)
C7—C1—C2—C3	−177.2 (2)	C10—C9—C8—C13	−0.7 (4)
C11—C10—C9—C8	0.1 (5)	C10—C9—C8—N1	−177.1 (3)
C4—C5—C6—C1	0.8 (5)	C7—N1—C8—C13	147.8 (2)
C2—C1—C6—C5	−1.0 (4)	C7—N1—C8—C9	−35.8 (4)
C7—C1—C6—C5	176.9 (3)	C9—C10—C11—C12	0.1 (5)
C5—C4—C3—C2	−0.2 (4)	C10—C11—C12—C13	0.3 (5)
C5—C4—C3—C14	179.0 (3)	C8—C13—C12—C11	−0.9 (4)
O1—C2—C3—C4	179.9 (2)	Br1—C13—C12—C11	−179.3 (2)

### Hydrogen-bond geometry (Å, °)

**Table:**

*D*—H···*A*	*D*—H	H···*A*	*D*···*A*	*D*—H···*A*
O1—H1···N1	0.89 (4)	1.81 (3)	2.611 (3)	149 (3)
